# Comment on: Simple implantation is safe for patients with totally implantable venous access ports

**DOI:** 10.1007/s00423-021-02164-2

**Published:** 2021-04-12

**Authors:** Felix Becker, Lennart A. Wurche, Martina Darscht, Andreas Pascher, Benjamin Struecker

**Affiliations:** Department of General, Visceral and Transplant Surgery, University Hospital Münster, Waldeyerstrasse 1, 48149 Münster, Germany

Dear Editor:

We are gratified to note the interest from Di Carlo et al. in our work and their comment related to our recent publication “Totally implantable venous access port insertion via open Seldinger approach of the internal jugular vein- a retrospective risk stratification of 500 consecutive patients” [1]. Di Carlo et al. have raised some important points in their letter to the editor and are happy to further discus them as well as to clear up all remaining misunderstandings.

Regarding their remark why the vessel wall isn’t incised but rather punctured, using the Seldinger technique, we have to state that we strongly believe that puncture and use of a guidewire are safer than a direct incision when using the internal jugular vein. The mentioned argument that this is risky without ultrasound guidance is incomprehensible since the vein is under direct vision in the surgical field. In addition, there appears to be misunderstanding regarding our figure as well as the text in the manuscript. Although stated otherwise in Di Carlo et al., we would like to assure that no clamp is used during any step of the procedure. The only instrument seen in the figure is a common wound retractor.

It is further mentioned that in cases of a failed attempt, using the cephalic vein cut-down technique, one should change to the coracobrachial, axillary, or external jugular vein. While we agree with the authors that this holds the inherent advantage of having an intraoperative exit strategy. However, our described technique is more suitable for patients with previous totally implantable venous access port (TIVAP) attempts or a complex vascular anatomy. In these cases, an alternative implantation side as well as a safe and reliable technique are necessary.

Lastly, it is stated that the reported complications (one puncture of the carotid artery and two cases of pneumothorax, 3 in 500 consecutive procedures) would not have happened if the procedure would have been finished surgically. Again, we would like to assure Di Carlo et. and the readership that all procedures were started and finished surgically. As described in the manuscript, our procedure had a completion rate of 100%.

In conclusion, our work closes a gap in the current knowledge concerning rescue TIVAP approaches by providing first data regarding safety, feasibility, and success for a modified procedure of TIVAP implantation via an open Seldinger approach of the internal jugular vein.
